# Genetically Predicted Circulating Level of C-Reactive Protein Is Not Associated With Prostate Cancer Risk

**DOI:** 10.3389/fonc.2020.545603

**Published:** 2020-10-14

**Authors:** Chiyu He, Yu Qian, Bin Liu, Shaoxue Yang, Ding Ye, Xiaohui Sun, Tianhui Chen, Yingying Mao

**Affiliations:** ^1^Second College of Clinical Medicine, Zhejiang Chinese Medical University, Hangzhou, China; ^2^School of Basic Medical Sciences, Zhejiang Chinese Medical University, Hangzhou, China; ^3^Department of Clinical Laboratory, Cancer Hospital of the University of Chinese Academy of Sciences (Zhejiang Cancer Hospital), Institute of Basic Medicine and Cancer (IBMC), Chinese Academy of Sciences, Hangzhou, China; ^4^Department of Cancer Prevention, Cancer Hospital of the University of Chinese Academy of Sciences (Zhejiang Cancer Hospital), Institute of Basic Medicine and Cancer (IBMC), Chinese Academy of Sciences, Hangzhou, China

**Keywords:** C-reactive protein, genome-wide association study, Mendelian randomization, prostate cancer, single-nucleotide polymorphisms

## Abstract

**Background:**

Inconsistent findings from observational studies have reported that C-reactive protein (CRP) is likely associated with risk of prostate cancer. Because conventional observational studies are susceptible to confounding and reverse causality, it remains unclear whether there is a causal relationship of CRP with risk of prostate cancer.

**Methods:**

In this study, we applied a two-sample Mendelian randomization (MR) approach to evaluate the potential causal association of circulating CRP levels with prostate cancer risk. Instrumental variables (IVs) and corresponding genetic association estimates for circulating CRP levels were obtained from a meta-analysis of genome-wide association studies (GWASs) including 204,402 participants of European descent. The genetic association estimates of these IVs with prostate cancer were obtained from a GWAS meta-analysis including 79,148 cases and 61,106 controls of European ancestry. The inverse-variance weighted (IVW) method was used as primary MR analyses, whereas in sensitivity analyses, MR-Egger regression, and MR pleiotropy residual sum and outlier (MR-PRESSO) test were used to assess the presence of pleiotropy. Odd ratio (OR) and 95% CI were calculated.

**Results:**

Overall, 58 single-nucleotide polymorphisms were used as instruments for circulating CRP levels. MR analysis suggested that genetically determined CRP levels were not associated with prostate cancer risk (OR 1.06, 95% CI 0.96 to 1.16) using the IVW method. Sensitivity analyses using alternative MR methods produced similar results (OR 1.00, 95% CI 0.93 to 1.08 for the weighted-median method; OR 1.02, 95% CI 0.95 to 1.08 for MR-PRESSO test). MR-Egger regression did not suggest evidence of directional pleiotropy (*P* = 0.25).

**Conclusion:**

Our study found that genetically predicted circulating CRP levels were not associated with prostate cancer risk, suggesting that CRP is unlikely to be a causal factor in the development of prostate cancer.

## Introduction

Prostate cancer is the second most frequently diagnosed cancer and the fifth leading cause of cancer death in men worldwide, with estimations of almost 1.3 million new cases and approximately 359,000 deaths in 2018 (1). Although age, ethnicity, family history of prostate cancer, and lifestyle such as smoking are established risk factors for prostate cancer, its etiology and pathogenesis remains to be fully elucidated (2).

In the past decades, chronic inflammation has been suggested to play a pivotal role in the development of prostate cancer (3). Because circulating C-reactive protein (CRP) is an important biomarker for low-grade chronic inflammation, the association of this biomarker with the incidence of prostate cancer has been investigated in several observational epidemiological studies. Some prospective studies found a positive association of CRP with risk of prostate cancer (4–6), but others did not provide evidence to support this relationship (7–11). Moreover, because conventional observational studies are susceptible to potential bias such as unmeasured confounders and reverse causality, it remains unclear whether the association of CRP with prostate cancer risk is causal or not.

Mendelian randomization (MR) analysis is a genetic epidemiological method which utilizes instrumental variables (IVs) such as single-nucleotide polymorphisms (SNPs) as proxies for a risk factor, and thus making causal inferences about a given exposure and an outcome (such as CRP and prostate cancer) (12). By taking advantage of the random assortment of genetic variants during gamete formation, MR studies are less vulnerable to reverse causality bias (12). Moreover, because the genetic variants are presumed to be distributed randomly at conception, they are generally uncorrelated with confounding factors such as lifestyle factor and socioeconomic position, and precede temporally both of the risk factors and the disease process (12). Therefore, in this study, we aimed to evaluate potential causal association of circulating CRP levels with risk of prostate cancer, using the two-sample MR study design.

## Materials and Methods

### Outcome Data Source

Summary level statistics of genetic association estimates for prostate cancer was obtained from a meta-analysis of genome-wide association studies (GWASs) including 79,148 cases and 61,106 controls of European ancestry conducted by the Prostate Cancer Association Group to Investigate Cancer-Associated Alterations in the Genome (PRACTICAL) Consortium. Full details of the study are available elsewhere (13) and in Supplementary Table 1. Briefly, a total of 79,148 cases with prostate cancer, and 61,106 cancer-free controls were involved. Each participating study was approved by the relevant ethics committee, and informed consent was obtained from all participants.

### Selection of Instrumental Variables

Single-nucleotide polymorphisms associated with circulating levels of CRP identified from a GWAS meta-analysis including 204,402 participants of European descent were used as IVs. Detailed information of this study is described elsewhere (14). Briefly, circulating levels of CRP (mg/L) were measured by using standard laboratory techniques, and the values were transformed by natural log. Individuals with autoimmune diseases, taking immune-modulating agents (if this information was available), or with CRP amounts 4 SD or more away from the mean were excluded from the analyses. All participating studies were approved by its institutional review board.

Overall, 58 SNPs from independent loci associated with circulating CRP levels at genome-wide significance threshold of *P* < 5 × 10^–8^ were included as IVs for the MR analysis, which explained about 7% variance of circulating CRP levels.

### Statistical Analysis

Statistical analyses were performed using MendelianRandomization and MR pleiotropy residual sum and outlier (MR-PRESSO) packages in R version 3.6.2. To assess the strength of the IVs and the potential influence of weak instrument bias, we estimated the F-statistics using the method previously described (15). After obtaining the effect estimates of the associations for individual CRP-associated SNPs with prostate cancer risk, we generated the ratio estimates and SE using the Wald ratio and the delta method, respectively (16). To assess the potential causal relationship of circulating levels of CRP with risk of prostate cancer, we combined the ratio estimates using the inverse-variance weighted (IVW) method in a random-effects model (16). To test the robustness of the causal estimate, we also performed the weighted-median method, which can provide a consistent estimate if less than 50% of the weights comes from invalid SNPs (17).

To assess the presence of pleiotropy, we performed MR-Egger regression and MR-PRESSO test. Specifically, in MR-Egger regression, if the *P* value for the MR-Egger intercept <0.05, it indicates that there exists directional pleiotropic effects (18). MR-PRESSO test can detect outliers and corrects for horizontal pleiotropic effects, as outlying SNPs are excluded from the IVs and the effect estimates are reassessed (19). In addition, we manually scanned each of the IVs used for their potential secondary phenotypes using the GWAS catalog (http://www.ebi.ac.uk/gwas, accessed on February 26, 2020) (20) and reran the MR analysis using the SNPs solely associated with circulating levels of CRP as IVs.

## Results

**FIGURE 1:**
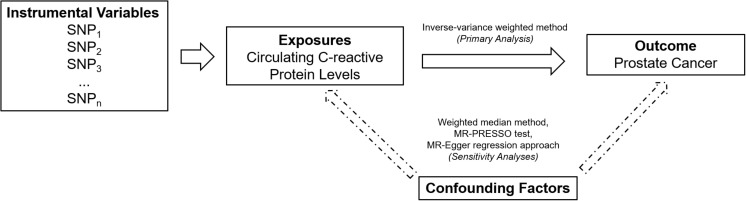
An overall design of the present study. MR, Mendelian randomization; MR-PRESSO, MR Pleiotropy RESidual Sum and Outlier; and SNP, single-nucleotide polymorphism.

**FIGURE 2:**
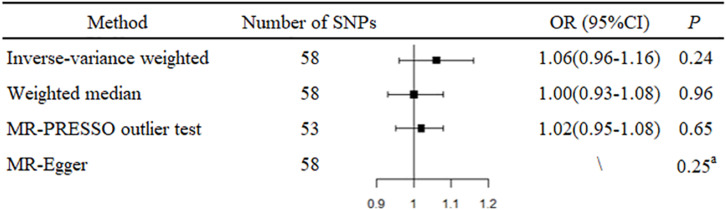
Forest plot of Mendelian randomization analysis for the associations of circulating C-reactive protein levels with risk of prostate cancer. MR, Mendelian randomization; MR-PRESSO test, MR Pleiotropy RESidual Sum and Outlier test; OR, odds ratio; and SNP, single-nucleotide polymorphism. ^a^*P* value of the intercept from MR-Egger regression analysis.

An overall design of the present study is shown in Figure 1. Detailed information about the 58 SNPs used as IVs for circulating levels of CRP and their association estimates with prostate cancer risk is listed in Supplementary Table 2. Among these SNPs, 14 were nominally associated with risk of prostate cancer (*P* < 0.05). MR analysis indicated that genetically predicted one-unit increase in the log-transformed circulating CRP levels were not associated with prostate cancer risk [odd ratio (OR) 1.06, 95% CI 0.96 to 1.16, and *P* = 0.24] using the IVW method based on a random-effects model (Figure 2). Similarly, the weighted-median method also showed that circulating levels of CRP were not causally associated with prostate cancer risk (OR 1.00, 95% CI 0.93 to 1.08, and *P* = 0.96).

Mendelian randomization-Egger regression analysis did not suggest evidence of potential directional pleiotropy (*P* value for intercept = 0.25). However, five outlier SNPs were identified using the MR-PRESSO test. The association between genetically predicted circulating CRP levels and prostate cancer risk did not change markedly after excluding these outlier SNPs (OR 1.02, 95% CI 0.95 to 1.08, and *P* = 0.64).

In addition, we manually scanned the GWAS catalog of each SNP used as IVs for their potential associations with secondary traits and found 25 potential pleiotropic SNPs (Supplementary Table 3). After exclusion of these SNPs, the MR effect estimate of circulating CRP levels on prostate cancer risk did not change essentially (OR 1.04, 95% CI 0.91 to 1.19, and *P* = 0.54 using the IVW method; Supplementary Table 4).

## Discussion

In this two-sample MR study, we found that genetically determined CRP levels were not associated with prostate cancer risk. Sensitivity analyses using alternative MR methods produced similar results. Collectively, these findings did not provide evidence for the causal effect of CRP on prostate cancer risk.

Recently, chronic inflammation has been hypothesized as an etiological factor for prostate cancer (3). One of the potential mechanisms that has been suggested is that highly reactive chemical compounds, such as singlet oxygen and superoxide, which are released from inflammatory cells, can damage many host epithelial cells by targeting their DNAs (21). In the process of replacing these cells, within the influence of the DNA-damaging agents, the risk of mutation increases, thereby promoting prostate cancer formation (3). CRP is an acute phase reactant which can rise rapidly in the circulation in response to inflammatory stimulus (22). Based on the evidence that potential inflammatory processes can affect the pathogenesis and the progression of cancer, CRP has been suggested to be an important biomarker for urological cancers including prostate cancer (23).

However, observational findings regarding the association of CRP with risk of prostate cancer were inconsistent. For instance, a cohort study including 8,471 Swedish participants found that men with high CRP levels (≥10 mg/L) had 29% (95% CI 7 to 56%) increased the odds of prostate cancer risk compared with those with low CRP level (<10 mg/L) (24). In contrast, another prospective study including 34,891 men reported that circulating levels of CRP were not associated with risk of prostate cancer [HR (hazard ratio) 0.87, 95% CI 0.65 to 1.16 for CRP concentrations 10–15 mg/L; HR 0.95, 95% CI 0.47 to 1.92 for CRP concentrations 15–25 mg/L; HR 1.35, 95% CI 0.74 to 1.92 for CRP concentrations 25–50 mg/L; and HR 0.90, 95% CI 0.37 to 2.19 for CRP concentrations >50 mg/L, compared with CRP concentrations <10 mg/L as the reference group] (10). A meta-analysis of five prospective cohort studies reported that circulating CRP levels were not associated with risk of prostate cancer (OR 1.06, 95% CI 0.97 to 1.16, and *P* = 0.83) (25), which was in line with findings from the present study.

The main strength of our study is that we used the MR approach which can minimize confounding and reverse causation bias inherent in conventional observational studies (12). With the availability of GWAS summary statistics, MR approach offers great opportunities to the etiological research of diseases, though the causal inference from MR study relies on three main assumptions as below. One assumption is that the IVs are associated with the exposure (26). Because the variance in circulating CRP levels explained by single SNP is limited, we used 58 independent SNPs associated with CRP at genome-wide significance as IVs to reduce the potential weak instrument bias. We calculated the corresponding F-statistics (265.2) and statistical power (83.8%), and found that our MR study had adequate power to detect moderate association and was unlikely to suffer from weak instrument bias. The second assumption is that the SNPs used as IVs are not associated with confounders that bias the observational epidemiological associations of the exposure with the outcome (26). Because the genetic alleles are presumed to be randomly allocated at conception, the confounding factors, such as socioeconomic and behavioral factors, are anticipated not to be associated with the allocation of genotype (12). Therefore, MR studies are less vulnerable to residual confounding compared with conventional epidemiological studies. The third assumption is that except for the association with the exposure of interest, the genetic variants used as IVs are not related to the outcome by other pathways (26). To assess the influence of potential pleiotropy on the causal effect estimate of the relationship between circulating CRP levels and risk of prostate cancer, we performed a series of sensitivity analyses, including the weighted-median method, MR-Egger regression, and MR-PRESSO test. We did not find evidence of directional pleiotropy, and all of these sensitivity analyses produced similar results. In addition, we scanned all the SNPs used as IVs for their secondary traits using the GWAS Catalog. After excluding 25 SNPs associated with other traits at genome-wide significance, we reran the MR analyses and obtained consistent results.

Our study also has limitations. First, our results may not be suitable to be extrapolated to the population with other ancestries because the ancestry of participants included in the summary statistics was restricted to European populations. However, this may also reduce the bias caused by population stratification. Another limitation is canalization, which means the compensatory developmental process will damp or buffer the effects of genetic variations on normal development (12). Hence, further longitudinal studies and MR analysis based on individual-level data as well as *in vivo* and *in vitro* functional studies are warranted to clarify the exact role of CRP in the development of prostate cancer.

## Conclusion

In summary, we found, for the first time using a two-sample MR approach with an adequate statistical power (including 79,148 cases and 61,106 controls), that genetically predicted circulating CRP levels were not associated with prostate cancer risk, suggesting that CRP is unlikely to be a causal factor in the development of prostate cancer.

## Data Availability Statement

The raw data supporting the conclusions of this article will be made available by the authors, without undue reservation.

## Author Contributions

TC and YM contributed conception and design of the study. YQ, CH, and SY organized the database. CH and YQ performed the statistical analysis. CH wrote the first draft of the article. SY, XS, and DY wrote sections of the article. All authors contributed to article revision, and read and approved the submitted version.

## Conflict of Interest

The authors declare that the research was conducted in the absence of any commercial or financial relationships that could be construed as a potential conflict of interest.
