# Supplementary material for: Genetically Predicted Circulating Level of C-Reactive Protein Is Not Associated With Prostate Cancer Risk
Source: Front Oncol. 2020 Oct 14;10:545603. doi: 10.3389/fonc.2020.545603 (PMC7591790; doi:10.3389/fonc.2020.545603)
Supplement: Supplementary file 1 [file Table_1.docx]

Supplementary Material

# Supplementary Tables

**Supplementary Table 1** Details of the genome-wide association studies and datasets used for the MR analysis.

**Supplementary Table 2** Characteristics of the genetic variants associated with circulating C-reactive protein and their effects on prostate cancer. Abbreviations: Chr, chromosome; SE, standard error; SNP, single nucleotide polymorphism.

**Supplementary Table 3** Associations between circulating C-reactive protein loci and other traits (from the GWAS Catalog)^a^. Abbreviations: Chr, chromosome; EA, effect allele; SNP, single nucleotide polymorphism. ^a^Traits associated with SNPs according to previous genome-wide association studies. Any ambiguities were not shown in this table.

**Supplementary Table 4** Effect estimates of the associations between circulating C-reactive protein and risk of prostate cancer after excluding potential pleiotropic SNPs. Abbreviations: CI, confidence interval; MR, Mendelian randomization; MR-PRESSO test, MR Pleiotropy RESidual Sum and Outlier test; OR, odds ratio; SNP, single nucleotide polymorphism. **P*-value of the intercept from MR Egger regression analysis.

# Funding acknowledgements related to PRACTICAL consortium

| **Supplementary Table 1. Details of the genome-wide association studies and datasets used for the MR analysis** | | | | |
| --- | --- | --- | --- | --- |
| Exposure or outcome | Study or consortium | Participants | Website | PMID |
| Prostate cancer | Prostate Cancer Association Group to Investigate Cancer-Associated Alterations in the Genome (PRACTICAL) Consortium | 79,148 cases and 61,106 controls of European ancestry | http://practical.icr.ac.uk/blog/?page_id=8164 | 29892016 |
| Circulating C-reactive protein | \ | 204,402 individuals of European ancestry | \ | 30388399 |

| **Supplementary** Table 2 Characteristics of the genetic variants associated with circulating C-reactive protein and their effects on prostate cancer | | | | | | | | | | | |
| --- | --- | --- | --- | --- | --- | --- | --- | --- | --- | --- | --- |
| SNP | Chr | Position | Gene | Effect Allele | SNP - C-reactive protein | | |  | SNP - Prostate cancer | | |
|  |  |  |  |  | β | SE | *P* |  | β | SE | *P* |
| rs10925027 | 1 | 247612562 | NLRP3 | T | 0.036 | 0.004 | 4.25E-21 |  | -0.008 | 0.008 | 0.3637 |
| rs1805096 | 1 | 66102257 | LEPR | G | 0.104 | 0.004 | 2.17E-183 |  | 0.003 | 0.008 | 0.7554 |
| rs2293476 | 1 | 40036847 | PABPC4 | C | 0.03 | 0.004 | 8.27E-13 |  | 0.020 | 0.010 | 0.04389 |
| rs2794520 | 1 | 159678816 | CRP | C | 0.182 | 0.004 | 4.17E-523 |  | -0.006 | 0.009 | 0.4977 |
| rs4129267 | 1 | 154426264 | IL6R | C | 0.088 | 0.004 | 1.2E-129 |  | -0.015 | 0.008 | 0.06772 |
| rs469772 | 1 | 91530305 | ZNF644 | T | -0.031 | 0.005 | 5.54E-12 |  | 0.020 | 0.010 | 0.05531 |
| rs75460349 | 1 | 27180088 | ZDHHC18 | A | 0.086 | 0.014 | 4.5E-10 |  | 0.022 | 0.026 | 0.4068 |
| rs1260326 | 2 | 27730940 | GCKR | T | 0.073 | 0.004 | 2.72E-92 |  | 0.022 | 0.008 | 0.00657 |
| rs12995480 | 2 | 629881 | TMEM18 | T | -0.031 | 0.005 | 1.24E-10 |  | 0.030 | 0.011 | 0.003964 |
| rs13409371 | 2 | 113838145 | IL1F10 | A | 0.048 | 0.004 | 5.07E-36 |  | 0.018 | 0.009 | 0.03685 |
| rs1441169 | 2 | 214033530 | IKZF2 | G | -0.025 | 0.004 | 2.27E-11 |  | -0.029 | 0.008 | 0.0002584 |
| rs4246598 | 2 | 88438050 | FABP1 | A | 0.022 | 0.004 | 5.11E-10 |  | 0.003 | 0.009 | 0.705 |
| rs9284725 | 2 | 102744854 | IL1R1 | C | 0.027 | 0.004 | 7.34E-11 |  | 0.010 | 0.010 | 0.286 |
| 3_47431869 | 3 | 47431869 | PTPN23 | D | 0.024 | 0.004 | 0.000000011 |  | 0.010 | 0.008 | 0.2333 |
| rs1514895 | 3 | 170705693 | EIF5A2 | A | -0.027 | 0.004 | 2.7E-09 |  | 0.027 | 0.009 | 0.003821 |
| rs2352975 | 3 | 49891885 | TRAIP | C | 0.025 | 0.004 | 6.43E-10 |  | -0.004 | 0.009 | 0.6764 |
| rs687339 | 3 | 135932359 | MSL2 | T | -0.03 | 0.005 | 2.8E-10 |  | 0.010 | 0.010 | 0.3156 |
| rs17658229 | 5 | 172191052 | DUSP1 | C | 0.056 | 0.01 | 5.5E-09 |  | -0.019 | 0.021 | 0.367 |
| rs12202641 | 6 | 116314634 | FRK | T | -0.023 | 0.004 | 3E-10 |  | -0.007 | 0.008 | 0.4155 |
| rs1490384 | 6 | 126851160 | C6orf173 | T | -0.025 | 0.004 | 2.65E-12 |  | 0.012 | 0.008 | 0.1361 |
| rs9271608 | 6 | 32591588 | HLA-DQA1 | G | 0.042 | 0.005 | 2.33E-17 |  | 0.065 | 0.010 | 4.71E-10 |
| rs9385532 | 6 | 130371227 | L3MBTL3 | T | -0.026 | 0.004 | 1.9E-11 |  | -0.034 | 0.009 | 7.68E-05 |
| rs13233571 | 7 | 72971231 | BCL7B | C | 0.057 | 0.005 | 2.95E-25 |  | 0.020 | 0.014 | 0.1373 |
| rs1880241 | 7 | 22759469 | IL6 | G | -0.028 | 0.004 | 8.41E-14 |  | 0.000 | 0.008 | 0.995 |
| rs2710804 | 7 | 36084529 | KIAA1706 | C | 0.021 | 0.004 | 0.000000013 |  | 0.005 | 0.008 | 0.5358 |
| rs7795281 | 7 | 74122854 | GTF2I | A | 0.028 | 0.005 | 0.000000031 |  | 0.028 | 0.010 | 0.003068 |
| rs1736060 | 8 | 11664738 | FDFT1 | T | 0.029 | 0.004 | 2.6E-13 |  | 0.015 | 0.008 | 0.0776 |
| rs2064009 | 8 | 117007850 | TRPS1 | C | -0.027 | 0.004 | 2.28E-14 |  | -0.007 | 0.008 | 0.4356 |
| rs2891677 | 8 | 126344208 | NSMCE2 | C | -0.02 | 0.004 | 1.59E-08 |  | 0.005 | 0.008 | 0.5029 |
| rs4841132 | 8 | 9183596 | PPP1R3B | G | 0.065 | 0.006 | 2E-25 |  | -0.005 | 0.014 | 0.7114 |
| rs643434 | 9 | 136142355 | ABO | A | 0.023 | 0.004 | 1.02E-09 |  | 0.008 | 0.008 | 0.341 |
| rs1051338 | 10 | 91007360 | LIPA | G | 0.024 | 0.004 | 2.27E-09 |  | -0.022 | 0.009 | 0.01397 |
| rs10832027 | 11 | 13357183 | ARNTL | G | -0.026 | 0.004 | 4.43E-12 |  | -0.014 | 0.009 | 0.09686 |
| rs10838687 | 11 | 47312892 | MADD | G | -0.031 | 0.004 | 9.12E-13 |  | 0.012 | 0.010 | 0.2278 |
| rs1582763 | 11 | 60021948 | MS4A4A | A | -0.022 | 0.004 | 2.37E-09 |  | 0.005 | 0.009 | 0.5853 |
| rs7121935 | 11 | 72496148 | STARD10 | A | -0.022 | 0.004 | 5.28E-09 |  | 0.003 | 0.008 | 0.7431 |
| rs10778215 | 12 | 103537266 | ASCL1 | T | 0.033 | 0.004 | 1.86E-20 |  | 0.002 | 0.008 | 0.7782 |
| rs11108056 | 12 | 95855385 | METAP2 | G | -0.028 | 0.004 | 5.42E-14 |  | 0.005 | 0.008 | 0.5473 |
| rs7310409 | 12 | 121424861 | HNF1A | G | 0.137 | 0.004 | 2.54E-299 |  | -0.013 | 0.008 | 0.1187 |
| rs112635299 | 14 | 94838142 | SERPINA1,SERPINA2 | T | -0.107 | 0.017 | 2.1E-10 |  | -0.126 | 0.030 | 2.48E-05 |
| rs2239222 | 14 | 73011885 | RGS6 | G | 0.035 | 0.004 | 9.87E-20 |  | -0.003 | 0.009 | 0.7023 |
| rs1189402 | 15 | 53728154 | ONECUT1 | A | 0.025 | 0.004 | 3.9E-09 |  | 0.006 | 0.009 | 0.474 |
| rs340005 | 15 | 60878030 | RORA | A | 0.03 | 0.004 | 1.01E-15 |  | 0.006 | 0.009 | 0.505 |
| rs4774590 | 15 | 51745277 | DMXL2 | A | -0.022 | 0.004 | 2.71E-08 |  | 0.000 | 0.008 | 0.9803 |
| rs10521222 | 16 | 51158710 | SALL1 | C | 0.104 | 0.011 | 2.06E-22 |  | -0.043 | 0.022 | 0.05179 |
| rs1558902 | 16 | 53803574 | FTO | A | 0.034 | 0.004 | 5.2E-20 |  | -0.016 | 0.008 | 0.0533 |
| 17_58001690 | 17 | 58001690 | RPS6KB1 | D | -0.026 | 0.004 | 9.5E-10 |  | 0.005 | 0.008 | 0.5804 |
| rs10512597 | 17 | 72699833 | CD300LF, RAB37 | T | -0.037 | 0.005 | 4.44E-14 |  | -0.023 | 0.010 | 0.02224 |
| rs178810 | 17 | 16097430 | NCOR1 | T | 0.02 | 0.004 | 2.95E-08 |  | 0.007 | 0.008 | 0.4074 |
| rs12960928 | 18 | 57897803 | MC4R | C | 0.024 | 0.004 | 1.91E-09 |  | -0.015 | 0.009 | 0.1105 |
| rs2852151 | 18 | 12841176 | PTPN2 | A | 0.025 | 0.004 | 1.36E-11 |  | 0.015 | 0.008 | 0.07263 |
| rs4092465 | 18 | 55080437 | ONECUT2 | A | -0.027 | 0.004 | 3.11E-10 |  | 0.010 | 0.009 | 0.2773 |
| rs4420638 | 19 | 45422946 | APOC1 | A | 0.229 | 0.006 | 1.23E-305 |  | 0.028 | 0.011 | 0.009704 |
| rs1800961 | 20 | 43042364 | HNF4A | C | 0.112 | 0.011 | 4.63E-23 |  | 0.015 | 0.022 | 0.4988 |
| rs2315008 | 20 | 62343956 | ZGPAT | T | -0.023 | 0.004 | 5.36E-10 |  | -0.078 | 0.009 | 1.98E-18 |
| rs2836878 | 21 | 40465534 | DSCR2 | G | 0.043 | 0.004 | 7.71E-26 |  | 0.018 | 0.010 | 0.0729 |
| rs6001193 | 22 | 39074737 | TOMM22 | G | -0.028 | 0.004 | 6.53E-14 |  | 0.000 | 0.008 | 0.9663 |
| rs9611441 | 22 | 41339367 | XPNPEP3 | C | -0.022 | 0.004 | 0.000000014 |  | -0.015 | 0.008 | 0.05327 |
| Abbreviations: Chr, chromosome; SE, standard error; SNP, single nucleotide polymorphism. | | | | | | | | | | | |

| **Supplementary Table 3** Associations between circulating C-reactive protein loci and other traits (from the GWAS catalog)^a^. | | | |
| --- | --- | --- | --- |
| SNP | Trait^a^ | *P*-value | PMID |
|  |  |  |  |
| rs75460349 | Systolic blood pressure | 1.00E-09 | 30578418 |
| rs2293476 | FEV/FEC ratio | 4.00E-20 | 30595370 |
|  | Drugs used in diabetes use measurement | 2.00E-08 | 31015401 |
| rs469772 | Total cholesterol measurement | 2.00E-08 | 27286809 |
| rs4129267 | Blood protein levels | 7.00E-1101 | 29875488 |
|  | Interleukin-6 receptor subunit alpha levels | 4.00E-58 | 25147954 |
|  | Fibrinogen | 6.00E-27 | 23969696 |
|  | Chronic inflammatory diseases (ankylosing spondylitis, Crohn's disease, psoriasis, primary sclerosing cholangitis, ulcerative colitis) | 9.00E-18 | 26974007 |
|  | Ankylosing spondylitis | 2.00E-15 | 23749187 |
|  | Asthma | 2.00E-08 | 21907864 |
| rs1260326 | Triglycerides | 1.00E-300 | 29507422 |
|  | Total cholesterol levels | 3.00E-160 | 30275531 |
|  | Blood metabolite ratios | 3.00E-148 | 24816252 |
|  | Urate levels | 3.00E-95 | 31578528 |
|  | Alcohol consumption | 2.00E-60 | 31358974 |
|  | White blood cell count | 4.00E-59 | 30595370 |
|  | Plasma lactate levels | 4.00E-52 | 26433129 |
|  | Gamma glutamyl transferase levels | 1.00E-48 | 29403010 |
|  | Gout | 4.00E-41 | 31578528 |
|  | Serum albumin level | 1.00E-40 | 29403010 |
|  | Lipid metabolism phenotypes | 1.00E-37 | 19936222 |
|  | Height | 1.00E-36 | 30595370 |
|  | Estimated glomerular filtration rate | 3.00E-36 | 31451708 |
|  | Bitter alcoholic beverage consumption | 2.00E-35 | 31046077 |
|  | Factor VII activity or levels | 8.00E-34 | 30642921 |
|  | Metabolite levels (small molecules and protein measures) | 7.00E-26 | 27005778 |
|  | Red cell distribution width | 1.00E-25 | 30595370 |
|  | Medication use (HMG CoA reductase inhibitors) | 1.00E-25 | 31015401 |
|  | Platelet count | 2.00E-25 | 27863252 |
|  | Type 2 diabetes | 5.00E-25 | 29632382 |
|  | High light scatter reticulocyte percentage of red cells | 4.00E-22 | 27863252 |
|  | Fat-free mass | 4.00E-22 | 30593698 |
|  | Plateletcrit | 1.00E-21 | 27863252 |
|  | Urinary sodium to creatinine ratio | 2.00E-21 | 30910378 |
|  | Serum total protein level | 4.00E-21 | 29403010 |
|  | Serum metabolite levels | 8.00E-20 | 31636271 |
|  | Neutrophil count | 1.00E-19 | 27863252 |
|  | Coffee consumption | 3.00E-19 | 31046077 |
|  | Serum uric acid levels | 8.00E-19 | 30993211 |
|  | Fasting blood glucose adjusted for BMI | 3.00E-18 | 25625282 |
|  | Granulocyte count | 7.00E-18 | 27863252 |
|  | Blood sugar levels | 2.00E-16 | 29403010 |
|  | Height | 2.00E-16 | 31562340 |
|  | Alcohol use disorder (consumption score) | 2.00E-16 | 30940813 |
|  | Gallstone disease | 2.00E-16 | 30504769 |
|  | Serum alpha1-antitrypsin levels | 3.00E-16 | 26174136 |
|  | Resting heart rate | 4.00E-16 | 27798624 |
|  | Inflammatory bowel disease | 1.00E-14 | 26192919 |
|  | Chronic kidney disease | 3.00E-14 | 20383146 |
|  | Liver enzyme levels (gamma-glutamyl transferase) | 4.00E-13 | 22001757 |
|  | Glycemic traits (pregnancy) | 6.00E-13 | 23903356 |
|  | Lymphocyte | 2.00E-12 | 27863252 |
|  | Urolithiasis | 5.00E-11 | 30975718 |
|  | Crohn's disease | 6.00E-11 | 28067908 |
|  | Two-hour glucose challenge | 3.00E-10 | 20081857 |
|  | Metabolic traits | 4.00E-10 | 19060910 |
|  | Hematocrit | 8.00E-10 | 27863252 |
|  | Branched-chain amino acid levels (Isoleucine) | 1.00E-09 | 27898682 |
|  | Hematological and biochemical traits | 4.00E-09 | 20139978 |
|  | Hypertriglyceridemia | 7.00E-09 | 20657596 |
|  | Low density lipoprotein cholesterol measurement | 2.00E-08 | 30926973 |
|  | Percent glycated albumin | 3.00E-08 | 29844224 |
| rs17658229 | Vertical cup-disc ratio | 8.00E-09 | 25241763 |
| rs1490384 | Height | 3.00E-166 | 30595370 |
|  | Brain region volumes | 2.00E-15 | 31676860 |
|  | Menarche (age at onset) | 1.00E-10 | 27182965 |
| rs2710804 | White blood cell count | 3.00E-19 | 30595370 |
|  | Fibrinogen levels | 3.00E-09 | 26561523 |
| rs13233571 | High density lipoprotein cholesterol levels | 5.00E-13 | 28334899 |
| rs4841132 | High density lipoprotein cholesterol levels x alcohol consumption (drinkers vs non-drinkers) interaction (2df) | 6.00E-114 | 30698716 |
|  | High density lipoprotein cholesterol | 1.00E-105 | 30275531 |
|  | High density lipoprotein cholesterol x physical activity interaction (2df test) | 2.00E-63 | 30670697 |
|  | Low density lipoprotein cholesterol levels x alcohol consumption (regular vs non-regular drinkers) interaction (2df) | 2.00E-48 | 30698716 |
|  | Total cholesterol levels | 2.00E-25 | 29507422 |
|  | Triglyceride levels x alcohol consumption (drinkers vs non-drinkers) interaction (2df) | 2.00E-15 | 30698716 |
|  | Glycemic traits (pregnancy) | 5.00E-15 | 23903356 |
|  | Fasting blood insulin | 6.00E-15 | 22581228 |
| rs643434 | End-stage coagulation | 1.00E-49 | 23381943 |
|  | Interleukin-6 measurement | 9.00E-25 | 22291609 |
| rs10832027 | Lipid traits (pleiotropy) (HIPO component 1) | 4.00E-12 | 30289880 |
| rs10838687 | Chronotype | 2.00E-13 | 30696823 |
|  | Proinsulin levels | 7.00E-12 | 21873549 |
| rs1582763 | Cerebrospinal fluid sTREM-2 level | 5.00E-21 | 31413141 |
|  | Alzheimer's disease in APOE e4- carriers | 2.00E-09 | 25778476 |
|  | Family history of Alzheimer's disease | 5.00E-09 | 30617256 |
| rs10778215 | Triglycerides | 5.00E-08 | 29507422 |
| rs7310409 | Liver enzyme levels (gamma-glutamyl transferase | 7.00E-45 | 22001757 |
| rs112635299 | Blood protein levels | 3.00E-33 | 29875488 |
|  | Alcohol consumption | 4.00E-12 | 31358974 |
|  | Coronary artery disease | 5.00E-10 | 29212778 |
|  | Post bronchodilator FEV1/FVC ratio | 2.00E-09 | 26634245 |
| rs340005 | Gamma glutamyl transferase levels in low alcohol consumption | 4.00E-09 | 30589442 |
| rs1558902 | Body mass index | 2.00E-223 | 28892062 |
|  | Waist circumference | 4.00E-101 | 25673412 |
|  | Obesity | 2.00E-81 | 23563607 |
|  | Weight | 2.00E-29 | 28552196 |
|  | Body fat percentage | 1.00E-25 | 26833246 |
|  | Red cell distribution width | 7.00E-25 | 30595370 |
|  | Hip circumference | 8.00E-20 | 28552196 |
|  | Childhood body mass index | 4.00E-14 | 26604143 |
|  | Menarche (age at onset) | 1.00E-12 | [27182965](https://www.ebi.ac.uk/gwas/publications/27182965) |
|  | Height adjusted BMI | 9.00E-10 | 25044758 |
|  | Glycated hemoglobin levels | 3.00E-08 | 28898252 |
| rs10512597 | Fibrinogen | 8.00E-11 | 20031577 |
| rs4092465 | Alcohol consumption (drinks per week) (MTAG) | 2.00E-09 | 30643251 |
| rs4420638 | Low density lipoprotein cholesterol levels low density lipoprotein cholesterol | 2.00E-178 | 24097068 |
|  | Total cholesterol levels | 1.00E-149 | 24097068 |
|  | Alzheimer's disease | 8.00E-149 | 22832961 |
|  | High density lipoprotein cholesterol levels | 2.00E-51 | 29507422 |
|  | Cerebrospinal fluid AB1-42 levels | 6.00E-42 | 28641921 |
|  | Longevity (90 years and older) | 3.00E-36 | 24688116 |
|  | Triglycerides | 8.00E-35 | 29507422 |
|  | Waist-hip ratio | 2.00E-34 | 30239722 |
|  | Lipoprotein-associated phospholipase A2 activity and mass | 5.00E-30 | 22003152 |
|  | Cerebrospinal fluid t-tau: AB1-42 ratio | 7.00E-29 | 30319691 |
|  | Cognitive decline | 4.00E-27 | 22054870 |
|  | Age-related disease endophenotypes | 2.00E-25 | 27790247 |
|  | Red cell distribution width | 2.00E-25 | 28957414 |
|  | Cingulate cortical amyloid beta load | 5.00E-21 | 26421299 |
|  | Cerebral amyloid deposition (PET imaging) | 7.00E-21 | 30361487 |
|  | Verbal declarative memory | 1.00E-16 | 25648963 |
|  | Platelet crit | 4.00E-16 | 27863252 |
|  | Blood protein levels | 3.00E-14 | 28240269 |
|  | Cerebrospinal fluid t-tau levels | 2.00E-13 | 28641921 |
|  | Body mass index (age>50) | 9.00E-12 | 26426971 |
|  | Cognitive impairment test score | 3.00E-11 | 30319691 |
|  | Coronary artery disease | 7.00E-11 | 26343387 |
|  | Body mass index x sex x age interaction (4df test) | 2.00E-10 | 26426971 |
|  | Waist-hip ratio | 5.00E-08 | 30575882 |
| rs1800961 | High density lipoprotein cholesterol levels x alcohol consumption (drinkers vs non-drinkers) interaction (2df) | 1.00E-71 | 30698716 |
|  | High density lipoprotein cholesterol measurement | 2.00E-65 | 30275531 |
|  | Gallstone disease | 6.00E-26 | 30504769 |
|  | Total cholesterol measurement | 1.00E-24 | 24097068 |
|  | Type 2 diabetes | 2.00E-22 | 30297969 |
|  | Red blood cell count | 6.00E-22 | 30595370 |
|  | Low density lipoprotein cholesterol levels x alcohol consumption (drinkers vs non-drinkers) interaction (2df) | 2.00E-12 | 30698716 |
|  | Neutrophil count | 6.00E-12 | 27863252 |
|  | Granulocyte count | 2.00E-11 | 27863252 |
|  | Fibrinogen levels | 1.00E-10 | 26561523 |
|  | Urate levels | 2.00E-10 | 31578528 |
|  | White blood cell count | 5.00E-10 | 30595370 |
|  | Hematocrit | 6.00E-10 | 27863252 |
| rs2315008 | Inflammatory bowel disease | 9.00E-15 | 18758464 |
| rs2836878 | Ulcerative colitis | 7.00E-53 | 26192919 |
|  | Inflammatory bowel disease | 5.00E-48 | 23128233 |
|  | Crohn's disease | 5.00E-15 | 26192919 |
|  | Triglycerides | 8.00E-09 | 30275531 |
| Abbreviations: Chr, chromosome; EA, effect allele; SNP, single nucleotide polymorphism. | | | |
| ^a^ Traits associated with SNP according to previous genome-wide association studies. Any ambiguities were not shown in this table. | | |  |

| **Supplementary Table 4** Effect estimates of the associations between circulating C-reactive protein and risk of prostate cancer after excluding potential pleiotropic SNPs. | | | | |
| --- | --- | --- | --- | --- |
| Method | Number of SNPs | OR | 95% CI | *P-*value |
| Inverse-variance weighted | 33 | 1.04 | 0.91-1.19 | 0.54 |
| Weighted median | 33 | 0.98 | 0.90-1.07 | 0.73 |
| MR-PRESSO test | 33 | 0.99 | 0.90-1.08 | 0.77 |
| MR-Egger | 33 | \ | \ | 0.30^*^ |
| Abbreviations: CI, confidence interval; MR, Mendelian randomization; MR-PRESSO test, MR Pleiotropy RESidual Sum and Outlier test; OR, odds ratio; SNP, single nucleotide polymorphism. **P*-value of the intercept from MR Egger regression analysis. | | | | |

**Funding acknowledgements related to PRACTICAL consortium**

The Prostate cancer genome-wide association analyses are supported by the Canadian Institutes of Health Research, European Commission’s Seventh Framework Programme grant agreement n° 223175 (HEALTH-F2-2009-223175), Cancer Research UK Grants C5047/A7357, C1287/A10118, C1287/A16563, C5047/A3354, C5047/A10692, C16913/A6135, and The National Institute of Health (NIH) Cancer Post-Cancer GWAS initiative grant: No. 1 U19 CA 148537-01 (the GAME-ON initiative).

We would also like to thank the following for funding support: The Institute of Cancer Research and The Everyman Campaign, The Prostate Cancer Research Foundation, Prostate Research Campaign UK (now PCUK), The Orchid Cancer Appeal, Rosetrees Trust, The National Cancer Research Network UK, The National Cancer Research Institute (NCRI) UK. We are grateful for support of NIHR funding to the NIHR Biomedical Research Centre at The Institute of Cancer Research and The Royal Marsden NHS Foundation Trust.

The Prostate Cancer Program of Cancer Council Victoria also acknowledge grant support from The National Health and Medical Research Council, Australia (126402, 209057, 251533, , 396414, 450104, 504700, 504702, 504715, 623204, 940394, 614296,), VicHealth, Cancer Council Victoria, The Prostate Cancer Foundation of Australia, The Whitten Foundation, PricewaterhouseCoopers, and Tattersall’s. EAO, DMK, and EMK acknowledge the Intramural Program of the National Human Genome Research Institute for their support.

Genotyping of the OncoArray was funded by the US National Institutes of Health (NIH) [U19 CA 148537 for ELucidating Loci Involved in Prostate cancer SuscEptibility (ELLIPSE) project and X01HG007492 to the Center for Inherited Disease Research (CIDR) under contract number HHSN268201200008I] and by Cancer Research UK grant A8197/A16565. Additional analytic support was provided by NIH NCI U01 CA188392 (PI: Schumacher).

Funding for the iCOGS infrastructure came from: the European Community’s Seventh Framework Programme under grant agreement n 223175 (HEALTH-F2-2009-223175) (COGS), Cancer Research UK (C1287/A10118, C1287/A 10710, C12292/A11174, C1281/A12014, C5047/A8384, C5047/A15007, C5047/A10692, C8197/A16565), the National Institutes of Health (CA128978) and Post-Cancer GWAS initiative (1U19 CA148537, 1U19 CA148065 and 1U19 CA148112 – the GAME-ON initiative), the Department of Defence (W81XWH-10-1-0341), the Canadian Institutes of Health Research (CIHR) for the CIHR Team in Familial Risks of Breast Cancer, Komen Foundation for the Cure, the Breast Cancer Research Foundation, and the Ovarian Cancer Research Fund.

The BPC3 was supported by the U.S. National Institutes of Health, National Cancer Institute (cooperative agreements U01-CA98233 to D.J.H., U01-CA98710 to S.M.G., U01-CA98216 toE.R., and U01-CA98758 to B.E.H., and Intramural Research Program of NIH/National Cancer Institute, Division of Cancer Epidemiology and Genetics).

CAPS GWAS study was supported by the Swedish Cancer Foundation (grant no 09-0677, 11-484, 12-823), the Cancer Risk Prediction Center (CRisP; www.crispcenter.org), a Linneus Centre (Contract ID 70867902) financed by the Swedish Research Council, Swedish Research Council (grant no K2010-70X-20430-04-3, 2014-2269)

PEGASUS was supported by the Intramural Research Program, Division of Cancer Epidemiology and Genetics, National Cancer Institute, National Institutes of Health.
